# Hilbert’s Early Metatheory Revisited

**DOI:** 10.1007/s10670-025-00959-z

**Published:** 2025-05-13

**Authors:** Eduardo N. Giovannini, Georg Schiemer

**Affiliations:** 1 CONICET/Universidad Nacional del Litoral, Paraje El Pozo, 3000 Santa Fe, Argentina; 2 University of Vienna, Universitätsstrasse 7, 1010 Vienna, Austria

## Abstract

The article offers a novel reconstruction of Hilbert’s early metatheory of formal axiomatics. His foundational work from the turn of the last century is often regarded as a central contribution to a “model-theoretic” viewpoint in modern logic and mathematics. The article will re-assess Hilbert’s role in the development of model theory by focusing on two aspects of his contributions to the axiomatic foundations of geometry and analysis. First, we examine Hilbert’s conception of mathematical theories and their interpretations; in particular, we argue that his early semantic views can be understood in terms of a notion of *translational isomorphism* between models of an axiomatic theory. Second, we offer a logical reconstruction of his consistency and independence results in geometry in terms of the notion of *interpretability* between theories.

## Introduction

David Hilbert’s early work on the foundations of mathematics is mainly known for its formal axiomatic approach. In his influential monograph *Foundations of Geometry* from 1899 [hereafter *Foundations*], he provided one of the first abstract axiomatizations of Euclidean geometry. In a similar manner, he laid down the first axiom system for the arithmetic of the real numbers in the classic investigation “On the concept of number” (1900). A central characteristic of these formative contributions to modern axiomatics was the role assigned to *metatheoretical* issues. In particular, Hilbert carried out systematic investigations of the independence and consistency of his axiom systems, and achieved novel metatheoretical results that contributed to building new solid foundations for these mathematical theories.

The methodology introduced by Hilbert to carry out these metatheoretical investigations is usually described as one of the starting points of *model–theoretic* thinking in mathematics. Here is the standard interpretation of his consistency and independence results: to prove the consistency of his axioms for Euclidean geometry, he constructed an analytic geometry over a sub-field of the real numbers and argued that all of the geometrical axioms are satisfied in this geometry. Similarly, to conclude that a particular axiom was independent from the other axioms, he provided an ‘analytic model’ in which all these axioms are satisfied, but the former fails to be valid. This understanding of Hilbert’s early metatheory reflects the consensus view in the scholarly literature. For instance, it has been defended in several places by Jaakko Hintikka, who claims that “there is no doubt that Hilbert’s *Foundations of Geometry* was one of the main gateways of model-theoretical thinking into twentieth-century logic and philosophy” (Hintikka, 1988, p. 6).^1^

While such a model-theoretic interpretation of Hilbert’s early axiomatic metatheory is widespread and constitutes the “received view,” in recent years alternative reconstructions of his foundational work have been proposed. For instance, Eder and Schiemer (2018) have pointed out that even though in his consistency and independence proofs in *Foundations* Hilbert appealed to several concepts (viz., ‘true’, ‘satisfaction’, and ‘validity’) that might seem model-theoretic to a modern reader, his use of these key concepts was notably ambiguous and not necessarily model-theoretic in spirit. Moreover, Dean (2020) has recently developed a careful reconstruction of Hilbert’s (meta)-geometrical results in terms of the *syntactic* notion of proof-theoretic interpretation.^2^ In summary, these recent works have revealed that the classification of Hilbert’s methodology as prototypically model-theoretic is debatable, and important conceptual issues call for further elucidation.

The aim of this paper is to contribute to this debate on the understanding of Hilbert’s early metatheory of formal axiomatics, in order to re-assess its role in the development of a model-theoretic conception of theories. In a nutshell, our main thesis will be this: beside the “received view,” an alternative logical reconstruction of Hilbert’s metatheoretic approach in terms of the modern notions of syntactic translations between languages and the interpretability between theories is feasible. This reconstruction is not only consistent with the scarce textual evidence in Hilbert’s *Foundations* and in related writings, but provides a more adequate explanation of his scattered remarks on the method of modeling in later writings. Moreover, the translation-based reconstruction of Hilbert’s metatheory also conforms, as we will show, with a common practice in nineteenth-century mathematics to conceive of the reinterpretation of theories in terms of translations between languages.

To defend this thesis, we will implement a twofold approach. On the one hand, we will examine Hilbert’s reflections on his novel metatheory of axiomatic systems, occurring in published works and in his notes for lectures on the foundations of mathematics in the period between 1898 and 1905. In particular, unpublished sources such as the notes of the lecture series *Logische Prinzipien des mathematischen Denkens* held in 1905 will provide a more refined historical picture of his early metatheory of axiom systems. On the other hand, we will take into account Hilbert’s retrospective discussions of his consistency and independence proofs in *Foundations*, in his work on the foundations of mathematics from the 1920s and early 1930s, particularly in Section §1 of *Grundlagen der Mathematik* (Hilbert & Bernays, 1934). This two-pronged approach will provide us with a more solid ground to reassess Hilbert’s conception of axiomatic theories.

Our discussion of Hilbert’s early metatheory in these works will focus on two central interpretative points. The first concerns his conceptual understanding of mathematical languages, of axiomatic theories expressed in them, and of their interpretations. We will argue that Hilbert’s semantic views at the time of *Foundations* are best explained by resorting to the idea of a *translation* between fully interpreted mathematical languages, rather than by the model-theoretic notion of the interpretation of a ‘formal’ language in different structures. In other words, Hilbert’s metatheoretical results in analysis and geometry can be logically reconstructed by the use of syntactic translations between *fully interpreted* mathematical languages, that is, languages that come with a rigid and intended interpretation. Moreover, we will argue that this account of Hilbert’s approach is more adequate than the received view in two respects: first, it is in line with Hilbert’s retrospective remarks on the “method or arithmetization” underlying his consistency and independence results; second, it conforms with the standard mathematical practice of using translations in modeling results at the time of *Foundations*.

As is well known, the term ‘translation’ was often employed by late nineteenth-century mathematicians to refer to the idea of ‘model’ construction in mathematics, although the exact logical nature of the notion was never clearly articulated. Two notable examples are Henri Poincaré’s (1891) ‘translation dictionary’ to construct a model of hyperbolic geometry within ordinary Euclidean geometry, and Richard Dedekind’s (1888) categoricity proof of the natural number system based on the idea of a ‘translation’ mapping. Moreover, Hallett (2010, 2012) has described Hilbert’s method of modeling in *Foundations* as appealing to the idea of translation, even though only in schematic terms. In this paper, we thus aim at clarifying this idea of mathematical translation by offering a more systematic logical explanation; particularly, we will distinguish between *two notions* of translations in Hilbert’s early formal axiomatics. Roughly, the first notion is characteristic of his work on the arithmetic of real numbers and consists of a mapping that connects languages of structurally similar grammatical and logical form. The second type of translation applies to languages of different signatures and is a key component of his independence and consistency proof in *Foundations*.

The second, closely related interpretative point concerns the understanding and role of *isomorphisms* (or isomorphic mappings) in Hilbert’s work from the turn of the last century. A dominant view in the literature is that, at this early stage, Hilbert did not yet have a ‘precise, general notion of isomorphism at his disposal’ (Awodey & Reck, 2002, p. 14). At the same time, his own remarks in published and unpublished writings indicate that he made systematic use of structure preserving mappings (or, in his terms, “reversible one-one transformations”) in his metatheoretic results. Based on this, we will show that Hilbert not only had clear and general notion of isomorphism at hand. His use of isomorphisms also allows us to further elucidate his conception of mathematical languages and theories. In particular, we will argue that Hilbert’s conception of a translation between fully interpreted languages depended on the construction of structure-preserving mappings between mathematical systems. Moreover, the close connection between isomorphisms and translations also suggest a novel logical reconstruction of Hilbert’s central metatheoretical results, namely the consistency and independence results in axiomatic geometry.

The structure of the article will be as follows. Section 2 outlines the received view of Hilbert’s early conception of axiomatic theories in the context of his work on analysis and geometry. In light of this, we will analyze how his understanding of mathematical languages and of isomorphisms between the models of a theory relates to the modern model-theoretic understanding. Section 3 then turns to a discussion of Dedekind’s view on theories in 1888. A main focus here will be on the use of “translational isomorphisms” in his proof of the categoricity of arithmetic. Based on this, we will outline a novel account of Hilbert’s conception of the interpretation of mathematical theories in different models in Sect. 4. Section 5 will then offer a logical reconstruction of Hilbert’s consistency proofs based on the notion of interpretability between theories. Section 6 contains a brief summary of our findings.

## Hilbert on Theories and Isomorphism

Hilbert’s foundational work from the turn of the last century contains axiomatic presentations of several theories of mathematics. More specifically, in his *Foundations* (1899) and in “On the concept of number” (1900), he presents a novel axiomatic account of theories that is usually characterized as *abstract* or *structural* in character. Hilbert’s “methodological turn” with respect to the classical or orthodox view of the axiomatic method is thereby understood as follows: axioms are no longer viewed as self-evident truths about a given mathematical domain, for instance, intuitive (Euclidean) space or the real number field. Rather, an axiom system functions as an implicit definition of the primitive vocabulary of a given theory, thereby specifying the meaning of these terms. Moreover, the meaning of the primitive terms is taken to be specified completely by the properties expressed in the axioms in question. Hence, an axiomatic theory in this structural sense is detached from any intuitive or pre-theoretical meaning of primitive vocabulary in question.

At the same time, it should be emphasized that Hilbert’s axiom systems for Euclidean geometry and for the arithmetic of real numbers are not yet *formal* in the modern sense of the term, or even formal in the sense of his later formalist program in the foundations of mathematics. That is, Hilbert’s systems were not yet formalized in a logical language with specified syntactic rules. The logical system at the basis of the axiom systems was not made explicit, and even more, he lacked a clear notion of derivability or syntactic consequence. Sieg and others therefore call this early view “structural axiomatics" (as opposed to the subsequent work on “formal axiomatics” in the 1920s) (Sieg, 2020).

At this early stage, Hilbert could explain the “abstract” character of his novel axiomatic approach in a very general and imprecise logical way. In particular, he often repeated two genuinely semantic ideas: (i) his axioms for Euclidean geometry and analysis lay down or establish certain mathematical relations between a series of fundamental concepts which can have multiple realizations or instantiations in different “system of objects”; (ii) the primitive terms of an axiomatic theory do no have a fixed reference and a *substantive* meaning, but admit different interpretations relative to such systems. Consider, for instance, the following apt description of Hilbert’s *Foundations* given by his later collaborator Paul Bernays:

According to this conception, the axioms are in no way judgments that can be said to be true or false; they have a sense only in the context of the whole axiom system. And even the axiom system as a whole does not constitute the statement of a truth; rather, the logical structure of axiomatic geometry in Hilbert’s sense, analogously to that of abstract group theory, is a purely hypothetical one. If there are anywhere in reality three systems of objects, as well as determinate relations between these objects, such that the axioms of geometry hold of them (this means that by an appropriate assignment of names to the objects and relations, the axioms turn into true statements), then all theorems of geometry hold of these objects and relationships as well. Thus the axiom system itself does not express something factual; rather, it presents only a possible form of a system of connections that must be investigated mathematically according to its internal properties. (Bernays, 1922, p. 95, Mancosu 1998, p. 192)

According to this view, Hilbert at the time of *Foundations* viewed axiom systems not as sets of statements expressing facts about a given domain, but rather as hypothetical “schemata” or conditions that can be interpreted in different models.

### The Received View

Given this account of the structural axiomatic method, what precisely was Hilbert’s early conception of theories? According to a prominent interpretative view developed by Hintikka, Hodges, and Demopoulos, among others, Hilbert’s account should be understood as a precursor of the modern model-theoretic conception of theories and languages. This concerns, in particular, the use of analytical models and model constructions in Hilbert’s consistency and independence proofs developed in *Foundations* and in related writings. Let us dub this historical interpretation the *received view* of Hilbert’s conception of theories.^3^ Demopoulos (1994), for instance, considers “Hilbert as standing very much at the beginning of this development towards model theory” (ibid., p. 213). More recently, regarding the metatheoretic results in *Foundations*, Dean (2020) holds that “Hilbert’s constructions are thus prototypical of what we would now call model-theoretic consistency proofs.” More explicitly, Dean takes Hilbert’s “technique of his geometric consistency proofs in a manner reminiscent of the modern notion of *model-theoretic interpretability*.” (ibid. p. 357)

Generally speaking, one can argue that that the received view incorporates three key theses (or conceptual assumptions) required for an adequate understanding of Hilbert’s account of mathematical theories:

(A) The *subject-matter* thesis: axioms specify or “define” classes of structures. The subject matter of mathematical theories are thus higher-order concepts or classes of (isomorphic) models, in modern terms.
(B) The *invariance* thesis: all models satisfying the axioms are equally acceptable as interpretations of a theory. In particular, isomorphic models with structurally similar properties are taken as equally valid.
(C) The *reinterpretation* thesis: the mathematical language of a theory is formal in the sense that the primitive non-logical terms do not have a fixed reference but can be freely (re-)interpreted in the different models.

Given these conceptual assumptions, several points of commentary are in order. First, related to thesis (A), a general assumption discussed in the literature on the received view is that Hilbert’s axiomatic theories function as “structural definitions" of types of systems or structures.^4^ A system, according to Hilbert, is understood similarly to our modern notion of a set-theoretic structure. It consists of a domain of objects, possibly containing some designated or distinguished objects, as well as relations and functions on that domain. This reading is explicitly endorsed by Hodges (2018), who takes our modern notion of set-theoretic structure to be rooted in the work of Hilbert and others:

Structures in this sense are an invention of the second half of the 19th century—for example Hilbert handled them freely in his *Grundlagen der Geometrie*. (...) Heinrich Weber and Hilbert spoke of ‘Systeme von Dingen,’ to distinguish from axiom systems. (ibid., pp. 439-440)

Structures in this model-theoretic sense of the term thus form the main constituents of Hilbert’s semantic account of theories. Given this, the notion of invariance or indifference to particular interpretations expressed in thesis (B) is then naturally cashed out in terms of the notion of isomorphism, understood as a structure-preserving mapping between systems or models. We will return to this point below.

Arguably the most characteristic assumption of the received view is thesis (C). Roughly put, the central claim here is that Hilbert understood the primitive vocabulary of these axiomatic theories in terms of non-logical constants that are freely re-interpretable in the different systems of the theory in question. More specifically, Demopoulos (1994) draws to a distinction (first developed in Hodges, 1985) between two types of logical-mathematical languages, namely “Frege-Peano languages” and “first-order languages of model theory.” Languages of the first kind are regimented “fragments of natural language” whose non-logical expressions (including proper names and predicates) all have a fixed meaning or interpretation. In contrast, languages of the second type contain non-logical constants that can be interpreted in different models or structures. As Hodges puts it, “[i]n themselves they don’t refer to any particular relations, functions or individuals, but we can give them references by applying the language to a particular structure or situation” (ibid., p. 144).^5^

The relevant point for our discussion is that both Hodges and Demopoulos (among others) suggest that Hilbert, in his work on the axiomatic foundations of mathematics of the turn of the last century, already endorsed an essentially modern, model-theoretic conception of the non-logical vocabulary. In particular, one can find a distinction in his axiomatic work that is constitutive for the modern account of languages. This is the distinction between schematically understood non-logical constants as “expressions with a determinate meaning, but with no fixed reference” and variables as “expressions lacking any meaning while admitting a conventionally stipulated reference (within a given ‘range’).” More importantly, according to this standard view, Hilbert’s work already witnesses the relativity of the interpretation of non-logical vocabulary in different models or structures. The reference of a constant expression, for instance, the primitive geometrical terms in *Foundations*, can be specified relative to different interpretations of the language. This view differs significantly from previous uses of “Frege-Peano languages,” where the non-logical constants come with a fixed interpretation. Statements expressed in such a language are evaluated semantically based on a notion of truth simpliciter (in some intended interpretation) and not in terms of the modern model-theoretic notion of “truth in a structure.”

### The Role of Isomorphisms

Does the received view adequately represent Hilbert’s views on the relation between mathematical languages, axiomatic theories, and their models at the time of *Foundations*? This is clearly a difficult interpretive question, given that Hilbert’s own remarks on methodological issues at the time remained rather scarce. Nevertheless, we propose in what follows that there is an alternative to the received view, which we take to yield a more adequate reconstruction of Hilbert’s position. Interestingly, the *invariance* thesis (B) mentioned above presents a good starting point for a novel assessment of his views. We have argued above that this thesis is usually cashed out in terms of the notion of isomorphisms between models. It is therefore not surprising that Hilbert’s study of the independence and consistency of axioms and axiom systems in geometry is often associated with the notion of structure-preserving mappings or isomorphisms. In fact, in an often-cited passage from a letter to Frege at the turn of the century, Hilbert explicitly addresses the link between the specification of systems based on structure-preserving transformations and his metatheoretic results:

But it is surely obvious that every theory is only a scaffolding or schema of concepts together with their necessary relations to one another, and that the basic elements can be thought of in any way one likes. [...] In other words: any theory can always be applied to infinitely many systems of basic elements. One only needs to apply a reversible one-one transformation and lay it down that the axioms shall be correspondingly the same for the transformed things. This circumstance is in fact frequently made use of, e.g., in the principle of duality, etc., and I have made use of it in my independence proofs. (Frege, 1980, pp. 40–41)

This passage contains an informal description of an axiomatic theory as a “schema of concepts” and of isomorphisms between their models in terms of “reversible one-one transformations.” Moreover, note that Hilbert directly connects the notion of isomorphism with his central metatheoretic results, namely independence proofs for axioms of geometry. (We will return to this point in section 5.) But what precisely was his understanding of an isomorphism at the time?

It should be mentioned that Hilbert did not have an explicit definition of the notion, neither in Hilbert (1899) nor in Hilbert (1900). This is not surprising since, according to Mancosu et al. (2009), the first use of a general notion of isomorphism between arbitrary systems can be found in Bôcher (1904) and in Huntington (1906). Nevertheless, one can get a clearer grasp of Hilbert’s informal understanding of the notion (as used in his work on the foundations of geometry and analysis) by consulting several passages from unpublished sources at the time. For instance, in the notes for his lecture course *Zahlbegriff und Quadratur des Kreises* (as given in the winter semester 1899/1900), Hilbert specifies a metatheoretic property of his axiom system of complete ordered field similar to the modern notion of categoricity in terms of structure-preserving mappings^6^:

They define uniquely [*eindeutig*] a system of things, i.e. if one has another system of things, which satisfies all the same axioms, then the things of the first system can be one-to-one reversibly mapped [*umkehrbar eindeutig abbildbar*] to things of the second system. (Hilbert, 1900, p. 42, our translation)

A similar reflection on the idea of isomorphism and “unique determination” can be found in a passage of the third volume of Hilbert’s *Wissenschaftliche Tagebücher*. This particular passage can be dated *ca.* 1901-1902:

A concept is called uniquely determined [eindeutig bestimmt] by the axioms if every other system of things which also satisfies the axioms can be one-to-one reversibly related to the first system of things such that all properties and relations of the things are preserved. (Cod. Ms. 600, III, p. 131, our translation)

These passages exhibit informal characterizations of the notion of an isomorphism as the existence of a bijective mapping between the domains of two mathematical systems that “preserves” their relational structure. In other words, Hilbert’s conception of isomorphism considers mappings between “systems” or models of the *axioms* of a mathematical theory. This shows that he, at the turn of the last century, already had a standard and general mathematical notion of an isomorphism as a structure-preserving mapping at hand. His notion essentially corresponds to the one officially introduced some years later in the works of Bôcher, Huntington, and Weyl (cf. Mancosu et al. (2009)).

**Fig. 1:**
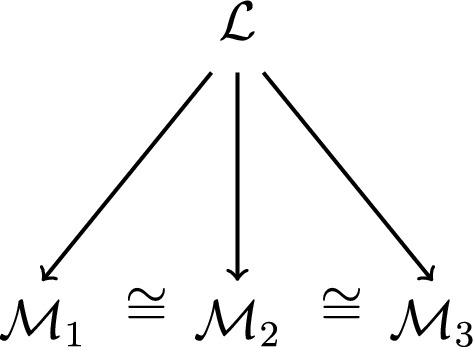
The model-theoretic conception of isomorphisms

At the same time, it is insightful to compare Hilbert’s mathematical notion of isomorphism with the modern model-theoretic definition. Roughly put, an isomorphism is specified in this context as a relation between two structures of a given formal language. More precisely, an isomorphism is usually defined as a bijective function between the domains of two structures ${\mathcal {M}}_1$ and ${\mathcal {M}}_2$ of a given language ${\mathcal {L}}$ (with a signature of non-logical constants) such that the function maps the semantic value of each constant expression in ${\mathcal {M}}_1$ to the semantic value of the expression in ${\mathcal {M}}_2$. Put differently, isomorphisms are specified in model theory as mappings that preserve the semantic interpretation of the non-logical vocabulary of a language in question (cf. Hodges (1993)). Given this account, two subtle conceptual differences to Hilbert’s understanding are noteworthy. First, the model-theoretic definition of isomorphisms is clearly *language-based* but theory-independent: it does not involve *models* of a given axiomatic theory but rather structures of a language with a given signature. Second, and more importantly, the model-theoretic notion critically presupposes that languages are formal in the modern sense of the term, i.e., that they are open to semantic *reinterpretation* in different structures. The following representation depicts this formal language-based conception of the concept of isomorphism (Fig. 1):

As we want to show below, this assumption of the semantic interpretability of formal languages as a key thesis of the received view is not required to explain Hilbert’s account. One can argue that his working notion of isomorphisms is in fact rooted in an alternative logical understanding of the relation between the mathematical languages, axiomatic theories formulated in them, and their semantic interpretations. In particular, we suggest that the idea of a *translation* between mathematical languages might be better suited for producing an alternative modern reconstruction of Hilbert’s early semantic views. Our contention is motivated by a reflection advanced by Hilbert in the lecture course *Logische Prinzipien des mathematischen Denkens* (Vorlesung, SS 1905)^7^; due to the significance of this reflection for our reconstruction, we will call it “the key passage.”

The context of this reflection is a discussion of the categoricity of his axiom system for the real numbers. More specifically, Hilbert presents a detailed argument that aims to show that *any* model of his axioms for complete ordered fields must be isomorphic to the “system of real numbers” genetically constructed, that is, as given by one of the standard set-theoretical infinitary constructions (e.g., upward closed sets of rationals or ‘Dedekind’s cuts’, or equivalence classes of fundamental sequences of rational numbers under an equivalence relation defined in terms of convergence). Immediately after reaching this result, Hilbert then stated a *categoricity statement* properly speaking, namely all models of the axioms for complete ordered fields must be isomorphic to one another (in modern terminology). Notably, to explain the notion of a structure-preserving mapping between any two systems of objects satisfying his axioms, Hilbert appeals to the idea of “translation”:

If we know of any system of things, that they satisfy our axiom-system, i.e., that the operations and relations between them hold for those properties, then for us they are none other than the real numbers. How they are named and *labeled, it does not matter at all*, because *the numbers have very different names in the different languages*. The essential is that between two of the systems which satisfy the axioms there is a one-to-one reversible relation between their things and operations, so that things $a, b, c, \dots$ of the first also uniquely correspond to such $a'$, $b'$, $c'$, $\dots$ of the second, such that every operation $a + b = c$ also corresponds to an $a' + b' = c'$ and so on. Then we say too, that the things are the same, namely the real numbers $a = a'$, $b = b'$, $\dots$, only the naming is different. $^{*}$[This] means a translation into another language. (Hilbert 1905, p. 21. Emphasis in original)

Hilbert’s explicit use of the notion of a ‘translation’ between languages is particularly interesting in this context, although certainly schematic. Accordingly, in what follows we will try to make more precise this suggestion of explaining the practice of ‘model’ construction in mathematics as based on translations between languages by proposing a more systematic logical reconstruction; specifically, we will argue that a particular type of translations, namely those induced by isomorphisms, played a key role in his early metatheory of formal axiomatics. To undertake this task, it will be beneficial first to examine briefly one of the most explicit and detailed references to the idea of translation of mathematical languages from this period, that is, Dedekind’s metatheoretical investigations in the essay *Was sind und was sollen die Zahlen?* of 1888 (henceforth WZ).

## Dedekind on Translational Isomorphism

The significance of Dedekind’s mathematical methodology for Hilbert’s early conception of axiomatics has been carefully investigated and stressed in the literature.^8^ Particularly, Dedekind’s abstract treatment of the arithmetic of natural numbers in WZ shares important common features with Hilbert’s axiomatic method by the turn of the nineteenth century. On the one hand, a fundamental aspect of Dedekind’s work was the focus on *abstract* concepts, that is, on higher-order concepts under which particular systems of mathematical objects fall. More importantly, these concepts were introduced or “created” by specifying a series of *characteristic conditions* or, in modern parlance, by laying down axiomatic or structural definitions. On the other hand, Dedekind’s structural characterization of the sequence of natural numbers was followed by the careful construction of structure-preserving mappings or isomorphisms between systems falling under this abstract concept, thus incorporating the metamathematical perspective as a key ingredient of his mathematical methodology.

As is well known, Dedekind defines natural number systems as *simply infinite systems* and proves several important “metatheoretical” results about arithmetic in this work.^9^ Simply infinite systems are specified axiomatically, in terms of a number of conditions that establish the basic structural properties of such systems. Here is Dedekind’s well-known definition, recast in modern terminology:

### Definition 1

A set *S* is said to be simply infinite if there exists a function *f* on *S* and an element $a \in S$ such that the following hold:

1. $f(S) \subseteq S$, i.e., *f* is a mapping from S into itself.
2. $a \notin f(S)$, i.e., *a* is not in the image of *S* under *f*.
3. $f(x)=f(y)$ implies $x=y$, i.e., *f* is a one–to–one function.
4. *S* is the smallest set containing *a* and closed under *f*, i.e., it is the intersection of all such sets.^10^

Given this concept of a natural number system, Theorem 132 in the book states that all simply infinite systems are similar (or, in modern terminology, isomorphic) to the intended system of natural numbers *N* and consequently (by Theorem 33) also to one another. In addition, Dedekind also proves that any theorem in number theory holds of any of the isomorphic systems in question. This is stated in the notable Remark 134:

### Remark134

(...) it is clear that every theorem regarding numbers, i.e., regarding the elements *n* of the simply infinite system *N* ordered by the mapping $\phi$ [...] possesses perfectly general validity for every other simply infinite system $\Omega$ ordered by a mapping $\theta$ and its elements $\nu$, and that the transfer from *N* to $\Omega$ (e.g., the translation of an arithmetical theorem from one language into another) is effected by the mapping $\psi$ [...] which transforms every element *n* of *N* into an element $\nu$ of $\Omega$, i.e., into $\psi (n)$. (Dedekind, 1888, p. 823)

The key notion mentioned in this passage is that of a translation between arithmetical theorems that is “effected” by the structure-preserving mapping $\psi$. In what follows, we will dub Dedekind’s notion of a translation induced by a canonical isomorphism a *translational isomorphism*.

### Canonical Languages and Translational Isomorphism

In the logical reconstruction of Dedekind’s metatheoretical result given in Sieg and Morris (2018), the informal idea of a “transfer from *N* to $\Omega$” is recast in the following way: let ${\mathcal {L}}$ be a pure second-order language without non-logical terms. A purely logical version of the conjunction of the four characteristic conditions of simply infinite systems can be expressed in this language. The resulting theory is logical in the sense that all non-logical primitive terms are systematically replaced by variables of a suitable type. Specifically, given Dedekind’s terminology, for a given system with domain *N*, a distinguished base element 1, and successor function $\phi$, the corresponding expressions in the pure language ${\mathcal {L}}$ are an existentially quantified individual variable *x*, an existentially quantified unary relation variable *X* and an existentially quantified function variable *f*.

As a result, one can think of the interpretation of the axiomatic theory in a particular model in Dedekind’s framework in terms of extensions of language ${\mathcal {L}}$ to arithmetical languages with a non-empty signature of primitive non-logical constants. Specifically, every ‘simply infinite system’ comes attached to a syntactic representation in a *canonical language*. Moreover, such languages have a fixed and rigid semantic interpretation in the sense that their non-logical terms refer to the objects in the corresponding number system. Compare Sieg and Morris (2018) on this approach:

Let *M* stand for the conjunction of the characteristic conditions. Replacing the variables *f* and *d* by particular symbols $\boldsymbol{\varphi }$, $\boldsymbol{1}$, and $\boldsymbol{\theta }$, $\boldsymbol{\omega }$, denoting $\varphi$, 1 and $\theta$, $\omega$ respectively, one obtains “canonical languages” $L^N$ and $L^{\Omega }$ for the particular simply infinite systems *N* (with $\varphi$ and 1) and $\Omega$ (with $\theta$ and $\omega$). (ibid., p. 273)

In this reconstruction of Dedekind’s approach, the method of interpreting the theory in a particular model, say in number system $(N, \varphi , 1)$, can thus be expressed in terms of the method of existential instantiation, that is, by the substitution of the existentially quantified variables *X*, *x*, and *f* by non-logical constants of a language such as $L^N$. Moreover, given Dedekind’s well-known result that any two such interpretations, say *N* and $\Omega$, are isomorphic, one can construct a translation function between the two corresponding languages that maps well-formed expressions of $L^N$ to those of $L^{\Omega }$.

Two details of this approach are relevant in the context of the present paper and are thus noteworthy. First, the two canonical languages in question are comparable in the sense of sharing a structurally similar signature, i.e., the same types of non-logical expressions. Hence, the translation in question preserves the logical form *and* the grammatical structure; in short, it presents an *isomorphic translation*.^11^ Compare again Sieg and Morris (2018):

If A is a statement in *L*, then $A^N$ and $A^{\Omega }$ are the statements in these canonical languages obtained from A by the obvious replacements; indeed, there is also a direct translation $\tau$ from $L^N$ into $L^{\Omega }$ associating $A^{\Omega }$ with $A^N$. (ibid, p. 273)

Second, given this account of a Dedekind translation between canonical languages, it should be emphasized that the translation function $\tau$ between languages $L^N$ and $L^{\Omega }$ is directly induced by the isomorphism $\psi$ between the corresponding structures $(N, \varphi , 1)$ and $(\Omega , \theta , \omega )$. To see this, recall that both arithmetical languages come with a rigid semantic interpretation. Thus, as Sieg and Morris (2018) point out, the symbols $\boldsymbol{\varphi }$, $\boldsymbol{1}$ in language $L^N$ denote the successor function $\varphi$ and the base element 1 in system $(N, \varphi , 1)$. Similarly, the symbols $\boldsymbol{\theta }$, $\boldsymbol{\omega }$ in language $L^\Omega$ denote the objects $\theta$, $\omega$ in system $(\Omega , \theta , \omega )$ respectively. Following their reconstruction, we can further assume that any number $n \in N$ has a name ${\textbf{n}}$ in $L^N$. We can thus define $g_{N}$ as a suitable interpretation function that maps each non-logical constant in $L^N$ to its corresponding object in system *N*. Similarly, let $g_{\Omega }$ be a suitable semantic interpretation function for language $L^{\Omega }$.

One can show then that the translation function $\tau$ can be constructed in terms of a “composition of mappings” of the interpretation functions and the isomorphism $\psi : N \rightarrow \Omega$ in the following way:

$$\begin{aligned} \tau = \overline{g_{\Omega }} \,\, \circ \,\, \psi \,\, \circ \,\, g_{N}, \end{aligned}$$

where $\overline{g_{\Omega }}$ presents the inverse of the interpretation function $g_{\Omega }$. The translation $\tau : L^N \rightarrow L^{\Omega }$ can be specified by the following recursive clauses:

(i) $$\tau (\boldsymbol{1}) = \overline{g_{\Omega }}(\psi (1)) = \boldsymbol{\omega }$$
(ii) $\tau (\boldsymbol{\varphi }({\textbf{n}})) = \overline{g_{\Omega }}(\theta \psi (n)) = \boldsymbol{\theta }(\tau ({\textbf{n}}))$, for any $n \in N$.

It is evident from the definition of this translation that the two canonical languages for arithmetic are “equivalent up to a translational isomorphism” (ibid, p. 274). Moreover, it should be noted that the translation induced by an isomorphism does not only show the structural similarity of the two canonical languages. It must also preserve either “true-value” or “theoremhood,” as Dedekind explicitly points out in Remark 134. Thus, the translation of formulas induced by function $\tau$ maps the parametrized axioms and theorems in $L^N$ to the corresponding parametrized axioms and theorems in $L^{\Omega }$.^12^

## A Reconstruction of Hilbert’s View

Returning now to Hilbert’s early views on theories and the practice of modeling in mathematics, we propose that a similar formal reconstruction to the one just outlined can be given.^13^ Specifically, we suggest that Hilbert’s account can be explained by analogy with Dedekind’s idea of translational isomorphisms. The starting point for our interpretation is again the “key passage” on isomorphism and translations from the lecture course *Logische Prinzipien* (1905), quoted in Sect. 2.2.

Hilbert’s informal remarks there can be recast in modern terms as follows: first, a formal axiom system or theory ${\mathbb {T}}$ can be formulated in a purely logical language ${\mathcal {L}}$. Thus, similarly to Dedekind’s case, an axiom system can be expressed in a purely logical form, based on the symbolization of the primitive expressions by variables of a suitable type. Second, every model of a theory ${\mathbb {T}}$ is described in a given *canonical language*. These canonical languages are obtained by extending ${\mathcal {L}}$ by constant expressions of the appropriate type. Assume that such mathematical languages came as fully interpreted both for Dedekind (as suggested in the above reconstruction) and for Hilbert. In particular, the primitive non-logical terms in their signatures all come with a fixed reference or semantic interpretation relative to a given domain.

We could then think of a canonical language as a tuple consisting of a formal language (with a specified signature) and a model-theoretic structure. The latter consists of a domain and an interpretation function that assigns distinguished objects, relations, and functions to the non-logical symbols of the language. Moreover, the substitution of the primitive variables of ${\mathbb {T}}$ by these constants renders the axiom system into a set of true propositions. As in the case of Dedekind’s WZ, one can think of the semantic interpretation of a theory in a particular model (or system of things) substitutionally, in terms of the substitution of non-logical constants of a canonical language for the primitive variables of theory ${\mathbb {T}}$. Finally, an isomorphism between two “models” of a theory ${\mathbb {T}}$ is *not* language-based in the model-theoretic sense. It is a mapping between the structures that induces such a translation between the corresponding canonical languages.

This proposal to formally recast Hilbert’s informal remarks on isomorphism and translation in 1905, in the context of the axiom system for the real numbers, is illustrated by the following diagram:

**Fig. 2:**
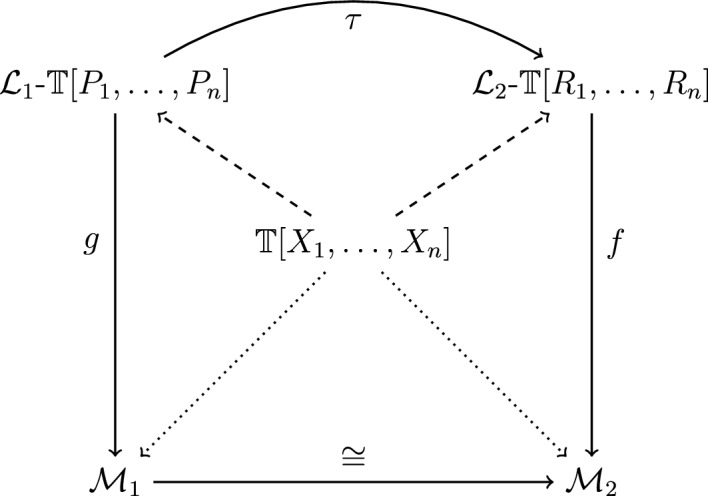
A Hilbertian account of theories and translational isomorphism

In this diagrammatic presentation, ${\mathbb {T}}[X_1,\dots , X_n]$ presents the purely logical formula expressing the axiomatic theory in question. Given the 1905 lecture, this could be the axiomatic theory of complete ordered fields first given in Hilbert (1900). In turn, ${\mathcal {L}}_1$-${\mathbb {T}}[P_1, \ldots , P_n]$ and ${\mathcal {L}}_2$-${\mathbb {T}}[R_1, \ldots , R_n]$ present two representations of this theory in the canonical languages ${\mathcal {L}}_1$ and ${\mathcal {L}}_2$ respectively. These languages come fully interpreted (based on the interpretation functions *g* and *f*) in two systems or models of the theory, namely ${\mathcal {M}}_1$ and ${\mathcal {M}}_2$. In light of Hilbert’s theory of analysis, the two models are both algebraic structures, namely complete ordered fields. A translation function $\tau$ between languages ${\mathcal {L}}_1$ and ${\mathcal {L}}_2$ is then induced by the isomorphism between ${\mathcal {M}}_1$ and ${\mathcal {M}}_2$ as well as by the interpretation functions *g* and *f* in the sense specified above.

Given this schematic account, a point of commentary is in order here. As indicated in the above diagram, a theory or axiom system can be expressed in a purely logical language. In contrast to the modern logical formalization, mathematical theories are not expressed in a language with a signature of non-logical primitive vocabulary. Rather, the primitives introduced by the axioms are symbolized by variables, say $X_1, \dots , X_n$ of an adequate type.^14^ Accordingly, the theory is expressed as a complex open formula ${\mathbb {T}}[X_1,\dots , X_n]$ where ${\mathbb {T}}$ stands for the conjunction of the axioms in question. We saw above that a similar view can be ascribed to Dedekind’s work. Now, admittedly, Hilbert’s reflections on axiomatic theories and (translational) isomorphism in “the key passage” of 1905 and in related passages from this period are too schematic to give full support to this logical reconstruction. However, it should be noted first that this approach conforms with the standard practice of formalizing axiom systems in logical work as late as the 1930s, for instance, by logicians such as Alfred Tarski and Rudolf Carnap. Second, and more importantly for our case, Hilbert himself adopted this approach to formalize axiom systems in his later proof-theoretic work, particularly in *Foundations of Mathematics* of 1934 co-authored with Paul Bernays.^15^

In section 1(a) of their book, titled “Formal axiomatics,” Hilbert and Bernays present a formalized version of an axiom system for Euclidean plane geometry. Unlike the system presented in *Foundations*, the system given in 1934 is one-sorted, describing only points as individuals. The axioms are formalized in a language of first-order logic and specify two primitive predicates, namely a ternary predicate *Gr*(*x*, *y*, *z*) standing for “the points *x*, *y*, *z* are collinear” and another ternary predicate *Zw*(*x*, *y*, *z*) standing for “a point *x* lies between points *y*, *z*.” Hilbert & Bernays present the axiom system as a complex logical formula *A*(*Gr*, *Zw*), based on the conjunction of all axioms. Moreover, they point out that in formal axiomatics, these primitive terms can be expressed as variables. They underline this point as follows:

In formal axiomatics, however, the basic relations are not conceived to be contentually determined from the outset; rather, they receive their determination only implicitly through the axioms. And any considerations within an axiomatic theory may make use only of those aspects of the basic relations that are explicitly formulated in the axioms. Thus, in axiomatic geometry, whenever we use names that correspond to intuitive geometry—such as “lie on” or “lie between”—this is just a concession to custom, and to ease the connection of the theory with the intuitive facts. Actually, however, in formal axiomatics, the basic relations play the role of *variable* predicates. (Hilbert & Bernays, 1934, p. 7)

Thus, the primitive predicates can be substituted by two ternary relation variables *R*(*x*, *y*, *z*) and *S*(*x*, *y*, *z*). Consequently, the theory is reformulated in terms of a purely logical version of the complex formula *A*(*Gr*, *Zw*), that is, *A*(*R*, *S*). As clarified by Hilbert & Bernays:

The axiom system consists of a requirement on two such predicates, which is expressed in the logical formula *A*(*R*, *S*), obtained from *A*(*Gr*, *Zw*) by replacing *Gr*(*x*, *y*, *z*) with *R*(*x*, *y*, *z*), and *Zw*(*x*, *y*, *z*) with *S*(*x*, *y*, *z*). (Hilbert & Bernays, 1934, p. 7)

This retrospective discussion of the landmark axiomatization of Euclidean geometry in *Foundations* thus reinforces the reconstruction developed here; specifically, it underscores the idea that an axiom system can be thereby expressed by a formula of pure predicate logic, in which the (mathematical) primitives are replaced by *variable* predicates. But what are the implications of this logical account for Hilbert’s understanding of the semantics of axiomatic theories, and in particular for the received view?

### Hilbert’s Early Semantics, Reassessed

The present reconstruction offers an alternative to the received view. It shows that Hilbert’s account of the semantics of axiomatic theories is not necessarily based on the notion of model-theoretic interpretability, that is, on the semantic interpretation of a theory expressed in a formal language in terms of different structures of that language. Rather, we saw that different models of a theory can be described in different *canonical languages*. Such languages are not viewed as formal in the modern sense, but rather as fully interpreted languages. The semantic interpretation of a theory in a system can then be recast purely *substitutionally*, based on the representation of the theory in fully interpreted canonical languages. Thus, the central conceptual difference to today’s model-theoretic account is this: instead of specifying the truth of the theory in a model in terms of the reinterpretation of a formal language (with a given mathematical signature), truth is specified in terms of the substitution of the primitive variables by interpreted constants of a canonical language. This syntactic substitution transforms the axiom system into a true proposition. In other words, a theory is true in a model if the sentence resulting from the systematic replacement of the primitive variables by non-logical constants turns out true. Thus, given the above diagram (Fig. 2), one can say that system ${\mathcal {M}}_1$ presents a model of the theory ${\mathbb {T}}[X_1,\dots , X_n]$ if ${\mathbb {T}}[P_1, \ldots , P_n]$ is a true sentence of canonical language ${\mathcal {L}}_1$. Notice that truth stands for truth simpliciter or truth in the intended interpretation of the canonical language in question. This simply means that the sentence ${\mathbb {T}}[P_1, \ldots , P_n]$ states a correct fact in the fixed interpretation of language ${\mathcal {L}}_1$. Interestingly, this substitutional account of truth of a theory in a structure was not exceptional at Hilbert’s time, but rather a common practice adopted by many mathematicians and logicians at the time.^16^

Another relevant point of commentary concerns Hilbert’s notion of isomorphism. As the passages quoted in section 2.2 show, the isomorphism between two models of a theory was understood *object-theoretically*, i.e., in terms of a bijective function that maps objects to objects and preserves the primitive relations defined through an axiom system. In Hilbert’s concrete example of real analysis discussed in the passage from the 1905 lecture, the bijection preserves the axiomatically specified properties of addition, multiplication, and so on. At the same time, we saw that there is no discussion of the semantic reinterpretation of mathematical languages but rather of a translation of one language into another language. As in Dedekind’s case, this syntactic translation between canonical languages is directly induced by the isomorphism between the two corresponding models. More specifically, given our schematic presentation of Hilbert’s translational isomorphism, the translation function $\tau$ between the closed terms and formulas of languages ${\mathcal {L}}_1$ and ${\mathcal {L}}_2$ can be constructed by the composition of the two interpretation functions *g* and *f* and the isomorphism $\cong$ in the following way:

$$\begin{aligned} \tau = {\overline{f}} \circ \cong \circ g, \end{aligned}$$

where ${\overline{f}}$ is simply the inverse of function *f*.

The fact that the translation is specified in this way has two important consequences. First, as we saw already in Dedekind’s treatment of arithmetic, it follows that $\tau$ maps primitive terms in the signature of ${\mathcal {L}}_1$ to primitive terms in ${\mathcal {L}}_2$. Consequently, the function also induces a translation of all axioms and theorems presented in canonical language ${\mathcal {L}}_1$ to the axioms and theorems presented in canonical language ${\mathcal {L}}_2$. Thus, one can say that the translation induced by an isomorphism preserves either “truth” or “theoremhood”. While this key aspect of translational isomorphism was addressed explicitly in Dedekind’s remark 134, the issue is (to the best of our knowledge) not raised in Hilbert’s work, neither in 1905 nor in related writings.^17^ Nevertheless, one can suggest two modern reconstructions of the fact that the isomorphism-induced translation is content-preserving. We could say that translation $\tau$ has the property of truth-preservation if the following holds:

$${\mathrm{For}}\;{\mathrm{all}}\;\varphi \in {\mathcal{L}}_{1} :{\mathrm{if}}\,{\mathcal{M}}_{1} { \vDash }\varphi\;{\mathrm{then}}\;{\mathcal {M}}_2 \models \varphi ^{\tau }$$

Alternatively, we say that $\tau$ is theorem preserving if, relative to a given formal system, we have

$$\text{For all} \,\,\varphi \in {\mathcal {L}}_1 : {\mathrm{if} \,\, {\mathcal {L}}_1} - {\mathbb {T}} \vdash \varphi \,\,{\mathrm{then}}\,\, {\mathcal {L}}_2 - {\mathbb {T}} \vdash \varphi ^{\tau }$$

To sum up, the present account of translational isomorphism between canonical languages offers a formal reconstruction of Hilbert’s informal remarks on the semantic interpretation of his axiom system for analysis, or more precisely, on the isomorphisms between complete ordered fields. Notably, this approach is closely in line with Dedekind’s axiomatic treatment of the isomorphism between natural number systems. It is also consistent with Hilbert’s own scattered methodological remarks in published and unpublished sources on the issue of model constructions, most importantly in his 1905 lecture. Finally, our reconstruction conforms with his retrospective reflections on the method of reinterpreting axiomatic theories from 1934. An interesting issue addressed in the next section is whether our reconstruction can also be applied to his consistency and independence results in *Foundations* (1899). While the notion of translational isomorphism fits very well with the reconstruction of Hilbert’s remarks in the context of axiomatic analysis, its application to the technique of model construction as executed in his metatheoretical work on geometry does not strike as immediately evident and deserves further scrutiny.

## Independence and Consistency in Geometry

In the last section, we have offered a reconstruction of Hilbert’s early conception of axiomatic theories which differs from the received view. The core element in our interpretation is that, from a conceptual point of view, Hilbert’s understanding of modeling in mathematics should not be explained by drawing on the modern notion of model-theoretic interpretation of formal languages in structures; instead, we propose that the idea of a syntactic translation between fully interpreted languages better accounts for his early metatheoretical views. Moreover, this practice of translation of mathematical languages is closely connected with Hilbert’s conception of isomorphisms, understood in terms of a bijective function that maps objects to objects and preserves the fundamental properties laid down in the axioms.

Hilbert’s metatheoretical work on geometry, especially his consistency and independence proofs in *Foundations* (1899), can also be associated with the notion of structure-preserving mappings and isomorphisms. Indeed, in the famous passage from the letter to Frege already quoted above (section 2.2), Hilbert seems to suggest that models of axiomatic theories can be specified based on structure-preserving transformations: “any theory can always be applied to infinitely many systems of basic elements. One only needs to apply a reversible one-one transformation and lay it down that the axioms shall be correspondingly the same for the transformed things.” Moreover, referring to this method, he explicitly adds here that “I have made use of it in my independence proofs” (Frege, 1980, pp. 40–41). Thus, according to Hilbert’s own assessment, his independence proofs in geometry are, methodologically speaking, based on the construction of models of a theory in terms of isomorphisms.

It is worth noting that, from the standpoint of the received view, Hilbert’s suggestion can be explained naturally in terms of the modern technique of *push-through constructions* in model theory. Very roughly, this is the method of constructing a new ${\mathcal {L}}$-structure ${\mathcal {N}}$ from a given ${\mathcal {L}}$-structure ${\mathcal {M}}$ and a bijective function $f: M \rightarrow N$ from the domain *M* of ${\mathcal {M}}$ to a set *N*. Given function *f*, one can easily define a new structure ${\mathcal {N}}$ with domain *N* that is isomorphic to ${\mathcal {M}}$. In particular, for a distinguished element $d \in M$ denoted by an individual constant in ${\mathcal {L}}$, let *f*(*d*) be its isomorphic copy in ${\mathcal {N}}$. Similarly, for any *n*-ary relation *R* in ${\mathcal {M}}$, an isomorphic copy of *R* in ${\mathcal {N}}$, say $R'$, can be defined as follows: $R' = \{\langle f(d_1), \dots , f(d_n) \rangle \mid \langle d_1, \dots , d_n \rangle \in R \}$.^18^

The connection between this elementary method of push-through constructions and the received view of Hilbert’s early metatheory is obvious; the former technique in modern model theory presupposes that mathematical languages are formal and open to reinterpretation. The isomorphic copies obtained via the push-through construction are all models of the same *formal* language. At the same time, this reading clearly conflicts with our reconstruction of Hilbert’s early semantic views, in which each model is tied to a canonical language and the practice of model variation is based on the syntactic translation between such languages. Given this, we propose a different logical reconstruction of the possible role of isomorphisms in Hilbert’s central metatheoretic results, namely the consistency and independence theorems in *Foundations*. To do this, let us first recall some basic facts about Hilbert’s axiomatization of Euclidean geometry and his construction of “analytic models.”

Hilbert’s original axiom system for Euclidean geometry consisted of 19 axioms divided in five groups: axioms of *Incidence* (I), *Betweennes* (II), *Congruence* (III); the Euclidean axiom of *parallels* (IV) and *Continuity* (V). The latter group included a standard version of the Archimedean axiom (AA). In subsequent editions, Hilbert also included a geometrical version of this axiom of completeness (AC) as an additional axiom of continuity,^19^ Although this axiom system was formulated in the ordinary geometrical language, it is standard now to formalize Hilbert’s language for Euclidean geometry (without AC) as a first order language ${\mathcal {L}}_H$ with two sorts of individual variables, namely point variables $A, B, C, \dots$ and line variables $l, m, n, \dots$. The signature of ${\mathcal {L}}_H$ consists of four primitive relations: a binary incidence relation *In*(*A*, *l*) (standing for “point *A* lies on line *l*”); a ternary betweenness relation *Bet*(*A*, *B*, *C*) (standing for “points *A*, *B*, *C* are collinear”); a quaternary relation $Con_{1}(A, B, C, D)$ (expressing that “line segments *AB* and *CD* are congruent”); finally, a 6-ary relation $Con_{2}(A, B, C, A', B', C')$ expressing the congruence between angles.^20^ Following Hartshorne (2000) and Baldwin (2018), we will call ‘Hilbert’s plane geometry’ ($\textsf{HP}$) the theory consisting of the axioms in group I–IV.

The basic technique first developed by Hilbert in the 1890 s to prove independence and consistency results is often described as the construction of “analytic models” of the geometrical axioms. In more precise terms, this technique consists in the specification of several different *coordinate geometries*. Roughly, the technique involves two main steps: first, the specification of a base number field; second, the definition of an (analytic) geometry over this numerical field. Hilbert provides such an example of the construction of a coordinate geometry in §9 of *Foundations*, where he advances a consistency proof of his axiom system. In this coordinate geometry, the base number field is the field $\Omega$ of all algebraic numbers obtained from 1 and the application in a finite number of times of the four rational operations of addition, subtraction, multiplication and division, and the fifth operation of $\sqrt{1 + \omega ^2}$, where $\omega$ denotes a constructible element in the field. In modern algebraic terminology, $\Omega$ is called a (minimal) Pythagorean field.

Next, Hilbert defines what he calls a “geometry,” that is, a system or relational structure over this field by providing definitions of the primitive geometrical predicates and relation symbols. This method corresponds to the standard construction in analytic geometry. As customary, Hilbert’s defines *points* as pairs of elements of the field $\Omega$ and *straight lines* as ratios (*u* : *v* : *w*) of three elements of such number field, not both $u=0$ and $v=0$. In other words, straight lines are identified with number triples pertaining to $\Omega ^3$. As for the relation of incidence, a point (*x*, *y*) lies on a line (*u* : *v* : *w*) if the following equation is satisfied:

$$\begin{aligned} ux + vy + w = 0 \end{aligned}$$

In *Foundations*, Hilbert describes very schematically how one can proceed to provide interpretations for the other primitive geometrical terms relative to this analytic model. Nevertheless, it will be useful for our subsequent discussion to present this briefly.^21^

Naturally, for the interpretation of the geometrical relation of betweenness one must impose additional structure on the field $\Omega$, that is, one must lay down additional conditions to obtain an *ordered Pythagorean field*. Then, one can define the geometrical relation of *betweenness* for points on a line as follows. Let $A=(x_1,y_1)$, $B=(x_2,y_2)$, $C=(x_3,y_3)$ be three distinct points on a line (*u* : *v* : *w*). We say that *B* is *between* *A* and *C* if

$$either\,x_1< x_2 < x_3\, or\, x_1> x_2 > x_3$$

The definition of congruence can be obtained as usual by means of the Euclidean distance function $\delta$, namely, the distance for two given points $A=(x_1,y_1)$, $B=(x_2,y_2)$ is equal to $\sqrt{(x_1 - x_2)^2 + (y_1 - y_2)^2}$. Now, since Hilbert is considering a sub-field of the reals numbers, i.e., a minimal Pythagorean field, which does not include certain square roots, it is convenient to use the “distance-squared” function, namely: $\delta ^2(A,B)=(x_1 - y_1)^2 + (x_2 - y_2)^2.$ Thus, one can define that the line segments *AB* and *CD* are *congruent* if the following equality holds:

$$\begin{aligned} \delta ^2(A,B)=\delta ^2(C,D). \end{aligned}$$

For reasons of space, we leave out the interpretation of the relation of congruence for angles. If the resulting geometry is constructed over a complete ordered field, then one obtains the “ordinary plane Cartesian geometry.”

This concise description of Hilbert’s specification of an analytic model of his geometrical axioms clearly indicates that his approach is not based on a push-through construction in the above sense, that is, on the determination of a bijection between domains that induces an isomorphism between models. Put differently, the specific use of isomorphisms or structure-preserving mappings outlined above does not seem to play a central role in his consistency and independence proofs. On the contrary, Hilbert’s remarks in Chapter II of *Foundations* rather suggest that he introduced new models based on the *definition* of the geometrical primitives in terms of (complex) formulas of the arithmetical language and, more generally, in the *interpretation* of the axioms for Euclidean geometry into the arithmetical theory of the field $\Omega$. Accordingly, the modern logical notions of interpretability between structures, as well as the concept of (relative) interpretability between theories, seem better candidates for reconstructing Hilbert’s metatheory. Crucially, both concepts are based on a purely syntactic notion of *translation between languages*. Let us examine this notion of translation and the related concepts of interpretability in more detail. We will show that these logical notions can be accommodated into our reconstructive framework and provide a better conceptual understanding of Hilbert’s early mathematical methodology.^22^

### Translations and Relative Interpretability

Our starting point will be the modern, purely syntactic notion of a *translation* of a language ${\mathcal {L}}_S$ (in which a theory ${\mathbb {S}}$ is formulated) into a language ${\mathcal {L}}_T$ (in which a theory ${\mathbb {T}}$ is formulated). More precisely, consider the following general definition of a translation:

#### Definition 2

An *n*-dimensional translation $\tau$ of ${\mathcal {L}}_S$ in ${\mathcal {L}}_T$ is given by the $L_T$-formulas

(i) $$\delta (x_1, \dots , x_n)$$
(ii) $\varphi _{R}({\bar{x}}_1, \dots , {\bar{x}}_m)$, for each relation symbol $R(x_1, \dots , x_m)$ of $L_S$ (where ${\bar{x}}_i$ present *n*-tuples of distinct variables)

such that

- $$(x=y)^\tau = ({\bar{x}} = {\bar{y}})$$
- $$(\lnot \varphi )^\tau = \lnot \varphi ^\tau$$
- $$(\varphi \wedge \psi )^\tau = \varphi ^\tau \wedge \psi ^\tau$$
- $$(\forall x \varphi )^\tau = \forall {\bar{x}} (\delta ({\bar{x}}) \rightarrow \varphi ^\tau )$$

For simplicity, we only consider relational languages in the present context, but the definition can be easily extended to languages with functional symbols. Schematically, a translation of a language ${\mathcal {L}}_S$ into a language ${\mathcal {L}}_T$ consists of two elements: i) a domain formula in $L_T$ that defines the domain of the interpreted structure; ii) for each relation symbol, a translation of atomic formulas formed by the primitive terms of ${\mathcal {L}}_S$ into (simple or complex) formulas of the language ${\mathcal {L}}_T$. The additional clauses impose that the translation function commutes with the logical connectives and quantifiers, thus preserving the logical form of sentences.^23^

Let us call $\textsf{PF}$ the theory of Pythagorean fields in Hilbert’s relative consistency proof. This theory is usually formalized in terms of a first-order language ${\mathcal {L}}_P$ with individual variables $x, y, z, \dots$, ranging over numbers and signature $\{ +, \times , 0, 1 \}$. Then, given the above notion of translation, one can think of an analytic model of Hilbert’s plane geometry $\textsf{HP}$ as based on a translation $\tau$ of the primitive vocabulary of ${\mathcal {L}}_H$ into the language of Pythagorean fields ${\mathcal {L}}_P$. Specifically, following Baldwin (2018), we can say that the relevant domain formula in ${\mathcal {L}}_P$ is the disjunction of the formulas $\delta _\tau ^P(x,y):x = x \wedge y=y$ and $\delta _\tau ^L(u,v,w): uvw\ne 0$, defining the two-sorted domain of points and non-trivial lines. Similarly, for each primitive relation (i.e., incidence, betweenness, segment congruence, angle congruence), atomic formulas of ${\mathcal {L}}_H$ are translated into (complex) formulas of ${\mathcal {L}}_P$. For instance, the atomic formula *I*(*A*, *l*) stating that a point *A* lies on a line *l* is translated into the equation $ux + vy + w = 0$ in language ${\mathcal {L}}_P$. Moreover, given that this translation $\tau$ also preserves the logical structure of the sentences translated, it follows that $\tau$ induces a translation of all axioms and theorems of Hilbert’s plane geometry into well-formed formulae of language ${\mathcal {L}}_P$. Let us dub $\tau (\textsf{HP})$ the translation of $\textsf{HP}$ into the language ${\mathcal {L}}_P$. Thus, $\tau (\textsf{HP})$ consists of complex, purely number-theoretic formulas given as values by the translation function $\tau$ for the geometrical axioms in question.

Given this framework, an important point needs to be made. The notion of translation described here differs from the Dedekindean notion of “translational isomorphism” introduced in the previous sections in one central aspect, for only the logical form but not the grammar of the languages in question is preserved. In addition, we saw that both in Dedekind (1888) and Hilbert (1905), translations between canonical languages are induced by an isomorphism between their corresponding interpretations. In contrast, in the present context, a translation connects languages with structurally different primitive vocabularies and is therefore not determined by such an object-theoretic mapping. We might refer to this general notion as a “Hilbert translation” between mathematical languages of different signatures. Nevertheless, both concepts correspond to a purely syntactic operation of the mapping of formulae between mathematical languages that preserves logical form; consequently, they can be equally applied to fully interpreted languages. We can thus think of the logical notion of translation presented above as a generalization of the Dedekindean idea of isomorphic translation, for the case of languages with different, ‘non-isomorphic’ signatures.

We can now use this account of the translation of the language of plane geometry into that of field theory to give a modern reconstruction of Hilbert’s relative consistency result by appealing to two related metatheoretic notions. The first one is the model-theoretic notion of an interpretation of a structure into another structure; more precisely, we will refer to the construction of *inner models*. The second one is that of an interpretation of one theory into another theory. Regarding the first, and given the notion of translation, one can construct an inner model, say ${{\mathcal {M}}}^\tau$, of a theory *S* in any given model ${{\mathcal {M}}}$ of theory ${\mathbb {T}}$:

#### Definition 3

(Inner models) An inner model ${{\mathcal {M}}}^\tau = \langle D^\tau , I^\tau \rangle$ of ${\mathbb {S}}$ is defined from ${{\mathcal {M}}} = \langle D, I \rangle$ of ${\mathbb {T}}$ by

1. $$D^\tau : = \lbrace {\bar{a}} \in D^n \mid {{\mathcal {M}}} \models \delta ({\bar{a}}) \rbrace$$
2. $I^\tau (R) = \lbrace \langle {\bar{a}}_1, \dots , {\bar{a}}_m \rangle \in (D^{n})^{m} \mid {{\mathcal {M}}} \models \varphi _{R}({\bar{a}}_1, \dots , {\bar{a}}_m) \rbrace$, for any *m*-ary relation symbol *R* of $L_S$.

Note that this method of inner model construction presupposes the modern semantic notion of *definability in a structure*.^24^ More precisely, the domain of objects and relations in the inner model ${{\mathcal {M}}}^\tau$ are *definable sets* in the model ${{\mathcal {M}}}$ and the defining formulas are precisely the *translation formulas* $\delta (x_1, \dots , x_n)$ and $\varphi _{R}({\bar{x}}_1, \dots , {\bar{x}}_m)$ in $L_T$. Definition 3 asserts that, given a translation $\tau$ of $L_S$ into $L_T$, we can construct from each $L_T$-structure ${{\mathcal {M}}}$ a certain $L_S$-structure ${{\mathcal {M}}}^\tau$, i.e., an inner model, by letting the domain of ${{\mathcal {M}}}^\tau$ be the set of objects in the domain of ${{\mathcal {M}}}$ that satisfy the domain formula $\delta (x)$, and by letting the interpretations of the primitive terms $R_i$ of $L_S$ be the *n*-tuples of objects in the domain of ${{\mathcal {M}}}$ that satisfy their translation formulas $\varphi _{R_i}(x_1,... x_n)$. The central aspect is that the inner model ${{\mathcal {M}}}^\tau$ constructed in this way from ${{\mathcal {M}}}$ is the structure that is induced by the translation $\tau$.

The notion of inner model allows to introduce the concept of interpretability between structures in the following way:

#### Definition 4

(Interpretability between structures) An ${\mathcal {L}}_{S}$ structure ${\mathcal {M}}$ is interpretable in a ${\mathcal {L}}_{T}$ structure ${\mathcal {N}}$ if ${\mathcal {M}}$ is isomorphic to an inner model ${\mathcal {N}}^{\tau }$ of ${\mathcal {N}}$.

Furthermore, based on the notion of a translation, one can specify a semantic version of the notion of an interpretation of theory ${\mathbb {S}}$ into theory ${\mathbb {T}}$:

#### Definition 5

(Interpretability between theories) $\tau$ is an interpretation of ${\mathbb {S}}$ in ${\mathbb {T}}$ if and only if

1. $\tau$ is a translation of $L_S$ in $L_T$ and
2. for every model ${{\mathcal {M}}} \models {\mathbb {T}}$, we have ${{\mathcal {M}}}^\tau \models {\mathbb {S}}$.
3. for any model ${{\mathcal {N}}} \models {\mathbb {S}}$ there is a ${{\mathcal {M}}} \models {\mathbb {T}}$ such that ${{\mathcal {M}}}^\tau$ is isomorphic to ${{\mathcal {N}}}$.^25^

How can the model-theoretic notions of inner models and interpretation be used to further analyze Hilbert’s consistency proofs in *Foundations*? As we saw in Sect.  4, it would be anachronistic to ascribe to Hilbert the modern model-theoretic conception of formal languages and theories. Rather, Hilbert likely understood languages in mathematics as canonical languages, that is, as languages that come with a rigid and intended interpretation. Moreover, systems or structures are usually introduced as models of particular theories. Thus, we can think of the standard interpretation of the geometrical language ${\mathcal {L}}_H$ as a structure of the form ${\mathcal {G}} = \langle P, L, In, Bet, Con_1, Con_2 \rangle$ that satisfies Hilbert’s plane geometry $\textsf{HP}$. Similarly, we can consider the language of Pythagorean fields ${\mathcal {L}}_P$ to describe a particular field investigated by Hilbert in this context. This is the Pythagorean field ${\mathcal {P}} = \langle \Omega , +, \times , 0, 1 \rangle$ already specified above; one can thus think of ${\mathcal {P}}$ as the standard model of the theory of Pythagorean fields $\textsf{PF}$.

Given the two canonical languages and the two associated structures ${\mathcal {G}}$ and ${\mathcal {P}}$, one can show that the specific translation function $\tau$ allows for the construction of an inner model of Hilbert’s geometrical theory $\textsf{HP}$ in the number-theoretic structure ${\mathcal {P}}$. Thus, in the context of Hilbert’s proof, his construction of a coordinate geometry can be explained as the construction of an inner model from a particular model (namely the intended model) of the theory of ordered Pythagorean fields. More specifically, given his definitions of the geometrical primitive vocabulary in terms of formulas of ${\mathcal {L}}_P$, one can construct a new structure, say $\tau ({\mathcal {G}}) = \langle P^{\tau }, L^{\tau }, In^{\tau }, Bet^{\tau }, Con_1^{\tau }, Con_2^{\tau } \rangle$, where each of the two domains and relations are given by definitions relative to set $\Omega$. Yet, one point must be emphasized here: while $\tau ({\mathcal {G}})$ is a structure of Hilbert’s geometrical signature, it is not, strictly speaking, an interpretation of the language ${\mathcal {L}}_H$. This is simply due to the fact that for Hilbert *anno* 1899, the idea of the semantic reinterpretation of a formal language in different structures was not yet at hand. Instead, we can think of structure $\tau ({\mathcal {G}})$ as the interpretation of another canonical language of geometry, say ${\mathcal {L}}_H'$, that also satisfies Hilbert’s axiom system (in the sense outlined in Sect. 4).

**Fig. 3:**
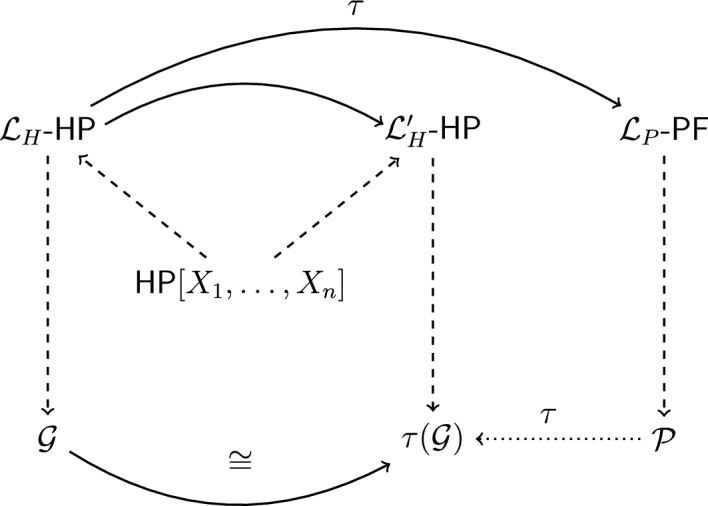
Hilbert translation in his construction of an analytic model $\tau ({\mathcal {G}})$

Given this set-up, we can now reconstruct the first step in Hilbert’s consistency proof, namely the construction of an analytic model of his theory of Euclidean planes, as an interpretability result between structures (as specified by Definition 4). In particular, his reasoning in §9 of *Foundations* suggests the result that the intended model of his theory of plane geometry is interpretable in the Pythagorean field ${\mathcal {P}}$. As Definition 5 shows, this is established by constructing an inner model $\tau ({\mathcal {G}})$ in ${\mathcal {P}}$ that is isomorphic to the intended geometrical structure ${\mathcal {G}}$. Given this modern reconstruction of Hilbert’s approach, two points should be emphasized here. First, note that the notion of isomorphism is again introduced here, as a relation between structures required for the specification of the relevant interpretability result. Nevertheless, this use of isomorphisms clearly differs from their use in push-through constructions of structures outlined above. Thus, our reconstruction suggests a different role of such mappings in Hilbert’s metatheory than suggested by the received view. Second, note that if one adopts the account of canonical languages specified in Sect. 4, then the structures ${\mathcal {G}}$ and $\tau ({\mathcal {G}})$ should not be viewed as isomorphic in the modern sense of the term, that is, as isomorphic structures of the same language ${\mathcal {L}}_H$. Rather, given Hilbert’s conceptual framework outlined above, the best way to characterize the notion of isomorphism here is as a structure-preserving mapping between two structures of distinct canonical languages. The following diagram illustrates the method of inner model constructions in the context of Hilbert’s consistency proofs (see Fig. 3).

What about the second step in his proof given in *Foundations*, that is, the demonstration that the theory of Hilbert’s planes is consistent relative to the consistency of the theory of Pythagorean fields? Based on the “analytic model” of his theory of geometry, Hilbert provided a relative proof of the consistency of the geometrical axioms (save the axiom of completeness). The general idea underlying this proof is described in “Mathematical Problems” (1900) as follows:

In geometry one can demonstrate the consistency of the axioms by constructing a suitable domain of numbers in a way such that there correspond relations between the numbers of this domain that are analogous to those of the geometrical axioms and that henceforth any contradiction in the consequences of the geometrical axioms would also have to be recognizable in the arithmetic of that number domain [...] The axioms of arithmetic are in essence nothing other than the well-known laws of calculation with addition of the axiom of continuity. I recently collected them in [Hilbert 1899]. (Hilbert, 1900b, pp. 264–265)

Thus, the essential feature of Hilbert’s consistency and independence proofs is that they are *relative*. In other words, these proofs are always formulated against the background of the theory of ordered fields in which the analytic models are constructed. Hilbert is clearly aware that we have to assume the consistency of the ‘arithmetic of the domain $\Omega$’ in his proof to establish the consistency of his axiom for geometry. The proof of the independence or consistency results for (fragments of) Euclidean geometry given in *Foundations* are therefore always formulated against the background of the theory of Pythagorean fields in which the analytical models are constructed. Now, Hilbert proves that the axioms of geometry are satisfied or valid under this interpretation and that this shows that the axioms of geometry are consistent, provided that the axioms for Pythagorean fields are also consistent. In the specific case discussed here, Hilbert’s consistency result is relative in the sense that the consistency of his theory of plane geometry is proven conditionally to the consistency of the theory of Pythagorean fields. In his own words: “Every contradiction in the consequences of the line and plane axioms I–IV, V, 1 would have to be detectable in the arithmetic of the field $\Omega$.” (Hilbert, 1971, p. 32).

Finally, as textual support for this reconstruction of Hilbert’s geometric metatheory, consider the following insightful remark by Hilbert and Bernays (1934), where the idea of a translation is explicitly mentioned:

Now, one usually treats this problem—both in geometry and the disciplines of physics—with the method of arithmetization: The objects of a theory are represented by numbers or systems of numbers and the basic relations by equations or inequations, such that, *on the basis of this translation*, the axioms of the theory turn out either as arithmetical identities or provable sentences (as in geometry) (...). This approach presupposes the validity of arithmetic, i.e.  the theory of real numbers (analysis). Hilbert & Bernays (1934, p. 3. Our emphasis)

The “method of arithmetization” outlined here seems to describe a close variant of the modern notion of interpretability between theories. First, a suitable translation function between the two languages allows one to “represent” all objects and primitive relations of one theory into number-theoretic objects and operations. Given such a translation, one can construct an inner model of the arithmetized theory in a certain number field. Second, it is shown that the arithmetical model satisfies the axioms of the theory in question. Alternatively, in proof-theoretic terms, the translation function preserves axioms and theoremhood. In the specific context of Hilbert’s consistency proofs in *Foundations*, this method ensures that Hilbert’s plane geometry can be interpreted in the arithmetic of the number field $\Omega$. Consequently, any inconsistency arising in the geometrical theory $\textsf{HP}$ would also have to arise already in the theory of Pythagorean fields $\textsf{PF}$.^26^

## Conclusion

The article gave a critical re-assessment and a logical reconstruction of Hilbert’s early contributions to the metatheory of axiomatic theories. As was mentioned in the introduction, Hilbert is often described in the scholarly literature as one of the precursors of modern model theory. More specifically, his axiomatic approach in *Foundations* of 1899 and in related work is characterized as based on a (proto-)model-theoretic conception of languages and theories. While there is certainly truth to such an assessment—Hilbert did, in fact, conceive of theories as being true or satisfied in different models in his metatheoretical results —a closer study shows that an alternative interpretation of his early views is feasible and consistent with his scattered methodological remarks. The main aim of this article was to outline a novel logical reconstruction of Hilbert’s views based on the notions of a syntactic translation between languages and the interpretability between theories.

In particular, starting from a discussion of the “received view” of Hilbert’s conception of languages and theories, we first surveyed different uses of the notion of isomorphisms as structure-preserving mappings between systems of axiomatic theories in his work. As was shown, Hilbert’s remarks on such mappings are closely linked to the notion of a translation between mathematical languages. Specifically, in the context of his work on real analysis, the relevant type of translations induced by an isomorphism connects canonical languages of the structurally similar and logical form. We dubbed this type of translations ‘Dedekind translations’, due to the close conceptual similarity to Dedekind’s preceding work in the foundations of arithmetic. A second, generalized form of translations between languages of different signatures, labelled ‘Hilbert translations’, was then used to reconstruct Hilbert’s metatheoretic work on axiomatic geometry, in particular, in his independence and consistency proofs presented in *Foundations*.

A general philosophical upshot of this survey is that Hilbert’s conception of theories likely differed from a modern model-theoretic perspective in important respects. Specifically, we take the main conceptual difference to be this: instead of thinking about axiom systems in terms of the modern concept of model-theoretic interpretability, that is, the interpretability of a theory expressed in a formal language in different structures, Hilbert’s work is best reconstructed as employing the concept of the relative interpretability between theories. As was shown in the article, his metatheoretic results both in analysis and Euclidean geometry can be viewed to depend on the systematic use of syntactic translations between fully interpreted, canonical languages that preserve the formal properties of the theories investigated. We therefore conclude with the suggestion that syntactic translations and the related concepts of interpretability between theories are the relevant tools to understand how Hilbert’s work anticipated a modern model-theoretic viewpoint.

## Footnotes

1 Compare Demopoulos (1994) and Blanchette (2017) for similar views of Hilbert’s approach.

2 Hallett (2010, 2012) has also advanced a reading of Hilbert’s early metatheory along these lines.

3 See, in particular, Hintikka (1988), Hodges (1985), and Demopoulos (1994) for different articulations of the received view.

4 On the role of axioms as describing or defining classes of models or structures, see Schlimm (2013).

5 According to Hodges, this feature of the reinterpretability of the non-logical expressions of a given language is required in the context of formalized mathematics: “(...) at least for mathematical purposes, we need non-logical constants whose interpretation is fixed in a structure but not fixed in the language” (ibid, p. 146). See Hallett (2010) for more details on the reinterpretability thesis in Hilbert’s work. Compare Eder (2021) for a rich historical study of the origins of model theory in nineteenth-century mathematics and the notion of a “reinterpretable language.”

6 A more detailed study of Hilbert’s early work on the categoricity of axiom systems, including a proof sketch of the categoricity of the theory of real number fields in Hilbert (1905), will be given in a separate article.

7 There are two existing versions—i.e., *Ausarbeitungen*—of this lecture course, one prepared by Hellinger (Hilbert 1905a) Born (Hilbert 1905b) and the other by Max Born. This lecture course is a particularly rich source of information, for it contains Hilbert’s most systematic discussion of the new axiomatic method corresponding to this early period of his foundational work. For more details on the content of this lecture course, see Corry (2004).

8 Ferreirós (2009), Klev (2011), and Sieg and Morris (2018) are important studies on the historical and conceptual relations between Dedekind’s work and Hilbert’s early axiomatics.

9 See, in particular, Sieg and Morris (2018), and Reck (2018) for detailed studies of Dedekind’s foundational work on arithmetic and analysis. In the following section, we will follow closely the reconstruction of Dedekind’s views given in Sieg and Morris (2018).

10 Dedekind (1888, §71). Cf. Awodey & Reck (2002, p. 6)

11 On the notions of logical form and grammatical structure, see Sect. 5 bellow.

12 Nevertheless, the exact nature of this characteristic feature of translations is a controversial topic in the literature. See again Sieg & Morris (2018, p. 274) on this point.

13 Hallett (2010, 2012) has suggested that Hilbert’s basic technique of modeling consisted, in strict terms, in “translating” one theory into another mathematical theory. We pursue this line of interpretation further by delivering a more systematic logical explanation based on new textual evidence as well as on the revaluation of known sources.

14 It is worth noting that Frege, in his famous controversy with Hilbert concerning the axiomatization of Euclidean geometry in *Foundations* clearly recognized that the geometrical primitives in the latter system could be formalized in a logical language using *variables*. As is well known, although Frege was very critical about this aspect of Hilbert’s new axiomatic method, he conveyed very precisely this view of mathematical axioms and primitives. For detailed studies of this aspect of the “Frege–Hilbert Controversy," see Resnik (1974) and Hallett (2012).

15 See, e.g., Mancosu (2010) and Schiemer and Reck (2013) for further details. Compare Sieg (2020) for a detailed study of the transition from Hilbert’s early work on “structural axiomatics” at the turn of the last century to the account of “formal axiomatics” underlying his proof-theoretic work in the 1920s and 1930 s. Finally, although it is widely acknowledged that the writing of *Grundlagen der Mathematik* (1934) was primarily the work of Bernays, it is also accepted that the ideas expressed in it accurately reflect Hilbert’s views.

16 See, in particular, Mancosu (2010) for a detailed study of this practice in logical work on formal axiomatics.

17 See again Sieg and Morris (2018) for a closer analysis of Dedekind’s remark about a “transfer from *N* to $\Omega$.”

18 Functions or operations induced by *f* can be specified analogously. See Button & Walsh (2018, p. 37) for further details.

19 In the *Festschrift* the Euclidean axiom of parallels was placed before the axioms of congruence. For a detailed analysis of the most important changes of Hilbert’s axiom system, and particularly the inclusion of the geometrical axiom of completeness, see Introduction to Chapter 5 of Hallett and Majer (2004).

20 See Hartshorne (2000), Baldwin (2018), and Dean (2020).

21 For a detailed presentation of the analytic models of Hilbert’s plane geometry see Hartshorne (2000), Baldwin (2018), Dean (2020).

22 In the recent scholarly literature on Hilbert’s early metatheory, it has been suggested that the relation between mathematical theories involved in his consistency and independence proofs can be made precise by means of semantic and syntactic notions of interpretation of one theory into another one. See, in particular, Eder and Schiemer (2018), Baldwin (2018), and Dean (2020). In this paper, we focus on the concept of translation between languages, which is at the base of these notions of logical interpretation of theories, and how this idea of translation is related to Hilbert’s early views on the semantics of mathematical languages.

23 For instance, the negation of a formula is translated into the negation of the translation of the formula, and similarly for the other truth-functional connectives. For quantifiers, there is a clause that stipulates that a quantified formula of ${\mathcal {L}}_S$ is translated to a quantified formula of ${\mathcal {L}}_T$ that is relativized to some ‘domain-formula’ $\delta (x)$. This relativization ensures that the quantifiers in the translated formula range over the objects of the domain imposed by $\delta (x)$ (cf. Eder & Schiemer (2018, p. 27)).

24 Let ${\mathcal {M}}$ be a structure and $n \ge 1$. A subset *X* of $M^n$ is called *L*-definable (with parameters ${\bar{b}}$) if there is an *L*-formula $\phi ({\bar{x}},{\bar{y}})$ and ${\bar{b}} \in M$ such that $X = \{{\bar{m}} \in M^n : {{\mathcal {M}}} \models \phi ({\bar{m}},{\bar{b}})\}.$ See Baldwin (2018, pp. 108-109) and Hodges (1993, §2.1) for further details.

25 A related, purely syntactic or proof-theoretic version of the notion of interpretability between theories holds that $\tau$ is an interpretation of theory ${\mathbb {S}}$ in ${\mathbb {T}}$ if and only if for any $\varphi \in {\mathcal {L}}_{S}$: if ${\mathbb {S}} \vdash \varphi$, then ${\mathbb {T}} \vdash \varphi ^{\tau }$. See, e.g., Hodges (1993) and Baldwin (2018) for further details.

26 See again Baldwin (2018) and Eder and Schiemer (2018) for a more detailed discussion of the different options to reconstruct Hilbert’s result.
